# Arylesterase Activity of Paraoxonase-1 in Serum and Cerebrospinal Fluid of Patients with Alzheimer’s Disease and Vascular Dementia

**DOI:** 10.3390/antiox9050456

**Published:** 2020-05-25

**Authors:** Arianna Romani, Alessandro Trentini, Wiesje M. van der Flier, Tiziana Bellini, Giovanni Zuliani, Carlo Cervellati, Charlotte E. Teunissen

**Affiliations:** 1 Department of Morphology, Surgery and Experimental Medicine and LTTA Centre, University of Ferrara, 44121 Ferrara, Italy; arianna.romani@unife.it (A.R.); giovanni.zuliani@unife.it (G.Z.); crvcrl@unife.it (C.C.); 2 Department of Biomedical and Specialist Surgical Sciences, Section of Medical Biochemistry, Molecular Biology and Genetics University of Ferrara, 44121 Ferrara, Italy; tiziana.bellini@unife.it; 3 Department of Clinical Chemistry, Neurochemistry Lab and Biobank, Amsterdam Neuroscience, Amsterdam UMC, Vrije Universiteit Amsterdam, 1081 HV Amsterdam, The Netherlands; wm.vdflier@amsterdamumc.nl (W.M.v.d.F.); c.teunissen@amsterdamumc.nl (C.E.T.); 4 Department of Epidemiology and Biostatistics, Vrije Universiteit Amsterdam, Amsterdam UMC, VU University Medical Center, De Boelelaan 1117, 1081 HV Amsterdam, The Netherlands

**Keywords:** paraoxonase-1, arylesterase activity, Alzheimer’s disease, vascular dementia

## Abstract

Background: It has been suggested that circulating Paraoxonase-1 (PON1) and apolipoprotein A1 (APOA1), which closely interacts with the antioxidant enzyme, could be implicated in Alzheimer’s disease (AD) and vascular dementia (VaD) development. This study aimed to evaluate PON1 changes in serum and cerebrospinal fluid (CSF) as evidence for its association with AD or VaD. Methods: Serum PON-arylesterase activity was measured in patients with AD, VaD, and CONTROLS distributed in two cohorts: Ferrara cohort (FC: *n* = 503, age = 74 years) and Amsterdam Dementia cohort (ADC: *n* = 71, age = 65 years). In the last cohort, CSF PON-arylesterase, CSF β-amyloid1-42, p-tau and t-tau, and imaging biomarkers were also measured. Results: AD and VaD patients of FC showed significantly lower levels of serum PON-arylesterase compared to CONTROLS, but this outcome was driven by older subjects (>71 years, *p* < 0.0001). In the younger ADC, a similar decreasing (but not significant) trend was observed in serum and CSF. Intriguingly, PON-arylesterase per APOA1 correlated with t-tau in AD group (*r* = −0.485, *p* = 0.002). Conclusion: These results suggest that decreased peripheral PON-arylesterase might be a specific feature of older AD/VaD patients. Moreover, we showed that PON-arylesterase/APOA1 is inversely related to neurodegeneration in AD patients, suggesting a prognostic usefulness of this composite parameter.

## 1. Introduction

Paraoxonase-1 (PON1) is classified as an accessory protein of high-density lipoprotein (HDL) and contributes to its anti-inflammatory and antioxidant functions, as well as to the ability to mediate reverse cholesterol transport [1,2,3]. In particular, through the synergistic cooperation with apolipoprotein A1 (Apo A1), the main protein constituent of HDL, PON1 protects this lipoprotein, as well as low-density lipoprotein (LDL), macrophages, and endothelial cells from oxidation [4,5]. These concerted actions are important components of a biological defensive mechanism against atherosclerosis and related diseases [2,6].

The pleiotropic role of PON1 might account for the documented association between low circulating levels of its activities and the risk for cardiovascular disease (CVD), but also pathologies affecting the brain, such as vascular dementia (VaD) and late-onset Alzheimer’s diseases (LOADs) [6,7,8,9,10,11]. It is now well accepted that oxidative stress (OxS) is both an early and late pathological hallmark of Alzheimer’s disease (AD) and VaD [12,13], not only in the brain but also in the systemic circulation. Hypercholesterolemia represents another condition associated with a high risk of VaD and AD [14]. Indeed, increasing evidence suggests that genes related to lipid metabolism and trafficking, such as APOE, CLU, SORL1, and PICALM, confer high susceptibility to AD [15,16]. Direct crosstalk becomes possible when the integrity of the blood brain barrier (BBB) barrier is compromised by vascular injury, as happens in VaD, and, although to a lesser extent, in AD [17]. Experimental evidence suggests that a high brain cholesterol content promotes amyloidogenic process and neurofibrillary tangles [15,18,19]. Relevant to the idea of an impact of PON1 in the central nervous system (CNS), we have recently found that the arylesterase activity (PON-arylesterase) is detectable in CSF [20]. This is intriguing since this fluid is in close interaction with brain tissue, and the evaluation of PON1 in CSF could give a glimpse into its possible role in the pathophysiology of brain disorders. However, whether the levels of CSF PON1 are altered in such diseases is still unknown.

The present study was conceived to validate the association between PON1 and AD or VaD. With these perspectives, serum PON-arylesterase activity was evaluated in a large cohort (*n* = 503), including cognitively normal CONTROLS and patients with LOAD or VaD, who were diagnosed by core clinical criteria. More importantly, we sought to confirm and extend the findings by measuring PON-arylesterase activity in paired serum and CSF of VaD, AD, and CONTROLS of an independent cohort (*n* = 71). This last cohort was also included to conduct an exploratory analysis about the possible influence of PON1 on classical AD biomarkers (β-amyloid (Aβ)1-42, p-tau, and t-tau). Therefore, only in this last cohort the clinical diagnosis was also supported by the combined use of conventional imaging (white matter hyperintensities, brain lacunes, and medial temporal lobe atrophy) and validated CSF AD biomarkers. 

## 2. Materials and Methods

### 2.1. Patients Selection

The study was conducted on two cohorts that will be referred as “Ferrara cohort (FC)” and “Amsterdam Dementia cohort (ADC)” for matter of clarity.

#### 2.1.1. Ferrara Cohort (FC)

Five hundred and three consecutive subjects referring to the Day Service for Cognitive Decline of Internal Medicine, S. Anna University Hospital, Ferrara, or of Casa della Sofferenza, San Giovanni Rotondo (Italy) were enrolled as previously described [9,21]. General characteristics, including age, gender, mini-mental state examination (MMSE), smoking, and comorbidities are shown in Table 1. For neuropsychological assessment, all patients were given a battery of tests as previously reported [22]. AD was diagnosed according to the National Institute on Aging–Alzheimer’s Association (NIA–AA) workgroups criteria [23]; of note, the large majority of these individuals were classified as late onset-AD (LOAD), reporting a disease onset at age ≥65 years (98%). The diagnosis of VaD was made following the National Institute of Neurological Disorders and Stroke and Association Internationale pour la Recherché et l’Enseignement en Neurosciences (NINDS-AIREN) criteria [24]. Apparently healthy individuals (CONTROLS) without evidence of dementia and without any functional disability attributable to cognitive impairment (MMSE range: 25–28) or other neurological diseases were also enrolled. 

The study on this cohort was in accordance with the Declaration of Helsinki and was approved by the local Ethic Committees (protocol n. 3877/DS and protocol n. 170579). Signed informed consent, which was written in compliance with local and national ethical guidelines, was obtained from each patient prior to the inclusion into the study. 

#### 2.1.2. Amsterdam Dementia Cohort (ADC)

This group included 71 subjects (general characteristics are shown in Table S2) referring to the memory clinic of the VU University Medical Center Amsterdam (VUmc) between November 2000 and August 2016 for extensive dementia screening that consisted of neurological, physical, and neuropsychological evaluation, biomarker analyses in CSF obtained by lumbar puncture, electroencephalography, and brain magnetic resonance imaging [25,26]. Subjects were labeled as “CONTROLS” upon multidisciplinary consensus when no abnormalities on clinical or cognitive testing were observed and criteria for MCI, dementia, and other medical conditions potentially causing cognitive decline were not met (i.e., no psychiatric diagnosis) [27,28]. The general selection criteria for this cohort included the availability of paired CSF and serum sample. The diagnostic criteria for VaD and AD are summarized as follows. The criteria for VaD patients was a clinical diagnosis of major vascular disease (VCD) based on the Vascular Behavioral and Cognitive Disorders (VASCOG) criteria [29] as detailed elsewhere [30]. Clinical diagnosis of AD was established by consensus in a multidisciplinary meeting according to the National Institute on Aging-Alzheimer’s Association criteria neuropsychological assessment [23].

The study was conducted in accordance with the Declaration of Helsinki. All subjects gave written informed consent for the use of clinical data for research purposes and the use of clinical data was approved by the local ethical review board. 

### 2.2. Serum and CSF Sampling

Venous blood was collected from FC’s subjects after overnight fasting. Each blood sample was then stored for one hour at room temperature and centrifuged (1200× *g* for 10 min) to obtain serum which was then divided into aliquots and stored at −80 °C until analysis.

CSF samples of the ADC were obtained by atraumatic lumbar puncture performed in the absence of contraindications. Within 2 h, CSF samples were centrifuged at 1800× *g* for 10 min at 4 °C, and the supernatant was biobanked at −80 °C until further analysis. Part of the CSF was centrifuged in the same way and stored at −20 °C for routine AD biomarker analysis as described below. Blood for serum separation was left to clot and processed in the same way as CSF. 

### 2.3. Assessment of CSF Aβ1-42, p-tau, and t-tau in CSF

CSF Aβ42, total tau and phosphorylated tau at Threonine 181 (p-tau) were measured with commercially available ELISA (Innotest-amyloid(1-42); Fujirebio, Ghent, Belgium) on a routine basis as described before [25,26]. CSF Abeta42 concentrations were adjusted for the drift in CSF biomarker analyses that occurred over the years and subsequently dichotomized as CSF amyloid abnormal (≤813 pg/mL) and amyloid normal (>813 pg/mL) [31].

### 2.4. Assessment of White Matter Hyperintensities (WMH), Brain Lacunes, and Medial Temporal Lobe Atrophy (MTA)

Scans were obtained on 4 different MRI scanners as previously described [32]. On the fluid-attenuated inversion recovery (FLAIR) sequence, WMH were assessed using the Fazekas scale (none, punctuate, early confluent, confluent; score 0–3). Medial temporal lobe atrophy (MTA) was rated (0–4) using oblique coronal reconstructions of T1-weighted gradient-echo volume sequences perpendicular to the long axis of the hippocampus. Global cortical atrophy was assessed on the axial FLAIR sequence (0–3). On both scales, maximal atrophy is represented by the highest score. Magnetic resonance imaging readings were dichotomized as the following: MBs, 0 vs. 1 or more; WMH, 0 or 1 vs. 2 or 3; lacunes, 0 vs. 1 or more; and MTA, (average score left and right side) 0 or 1 vs. 2 or more.

### 2.5. Apo A1 and PON-Arylesterase in Serum and CSF Samples 

Serum PON-arylesterase were measured by UV-VIS spectrophotometric assays in a 96-well plate format by using a Tecan Infinite M200 microplate reader (Tecan group Ltd., Menndorf, Switzerland).

Arylesterase activity was assessed by adding 10 μL of serum (diluted 1:25 with buffer) or 10 μL of neat CSF to 190 or 90 μL, respectively, of reaction mixture including 1 mM phenylacetate and 0.9 mM CaCl_2_ dissolved in 9 mM Tris-HCl, pH 8 [33]. A molar extinction coefficient of 1310 M^−1^ cm^−1^ was used for the calculation of enzyme activity, expressed in kilo unit per liter. One unit of arylesterase activity accounts for 1 µmol of phenol produced in a minute under the conditions of the assay.

Apo A1 was determined by Immunoturbidimetric method [34] by Central laboratory of S. Anna University Hospital, Ferrara.

### 2.6. Statistical Analysis

Data analysis was performed using SPSS Statistics for Windows, version 18.0 (SPSS, Inc., Chicago, IL, USA). Chi-square tests were used to compare differences in categorical variables. Continuous variables were first checked for normal distribution using Kolmogorov–Smirnov test. When not normal, log 10 transformation was applied to achieve normality. Group comparisons were performed using one-way analysis of variance (ANOVA) with Sidak *post-hoc* test. Possible confounding effects of covariates on the between-group differences were checked by analysis of covariance (ANCOVA). Simple correlation analyses were performed using Pearson’s tests after log 10 transformation for not normally distributed variable in order to approach a normal distribution. To check the robustness of the associations, the 95% confidence interval for the correlation coefficient was bootstrapped with 1000 samplings by using a bias-corrected and accelerated (BCa) approach. Multiple regression analysis was performed to check whether the observed correlations were independent of potential confounding factors such as age, sex, smoking habit, hypertension, and diabetes. Two-tailed probability values *p* < 0.05 were considered statistically significant.

## 3. Results

### 3.1. Population Characteristics

As shown in Table 1, FC patients that were older than CONTROLS (*p* < 0.05) presented with a lower MMSE score (*p* < 0.05), and VaD patients had a higher prevalence of diabetes than both CONTROLS and AD (*p* < 0.05 for both comparisons). On the contrary, the only differences found in ADC were on female prevalence (lower in VaD than CONTROLS, *p* < 0.05), hypertension (higher in VaD than CONTROLS, *p* < 0.05), and MMSE score, that was significantly lower in both VaD and AD subjects than CONTROLS (*p* < 0.05). As expected, AD patients had higher CSF levels of Aβ1-42, p-tau, and t-tau compared with the other two groups (Supplementary Table S1). In addition, WMLs and MTA were more frequent in AD and VaD, respectively, compared to the other groups; lacunes were almost solely present in VaD.

Of note, the FC and ADC showed significant differences with respect to age (all ADC subjects were significantly younger than those in FC, see Table 1), MMSE (CONTROLS and VaD patients in ADC had higher values, *p* < 0.05), prevalence of women (AD group in ADC had a lower frequency of females, *p* < 0.05), and hypertension (there was a lower prevalence of hypertension in CONTROLS and AD in Amsterdam cohort, *p* < 0.05).

### 3.2. Serum PON-Arylesterase Activity in FC 

**Figure 1:**
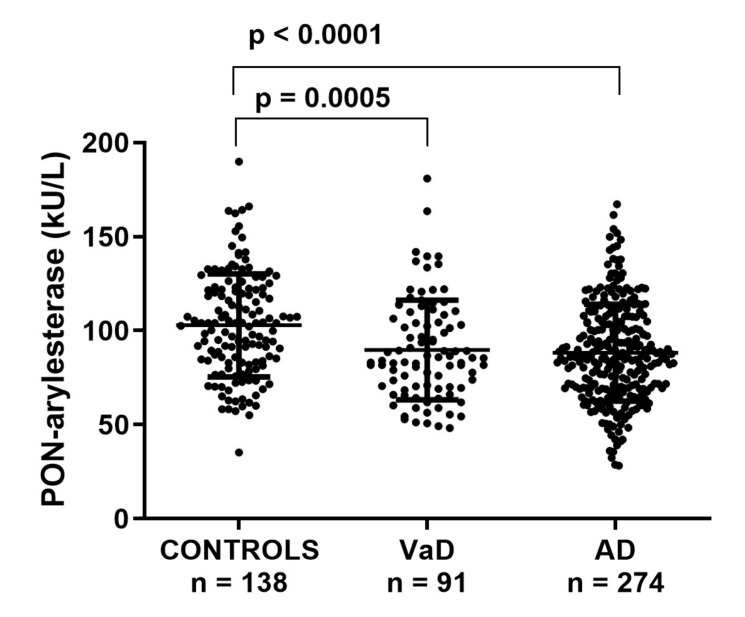
Scatter plots displaying serum PON-arylesterase activity in CONTROLS (*n* = 138), VaD (*n* = 91), and AD (*n* = 274) patients, within FC. Statistical analysis was performed using ANOVA followed by Sidak *post-hoc* test. Abbreviations: VaD, vascular dementia; AD, Alzheimer’s disease, PON, Paraoxonase-1

First, we checked for differences in serum PON-arylesterase levels within the groups in the FC. PON-arylesterase activity was significantly lower in AD (mean ± SD: 88 ± 26 kU/L) or VaD (87 ± 25 kU/L) compared to CONTROLS (101 ± 27 kU/L, *p* < 0.001 for all differences) (Figure 1). In the whole cohort, women had higher PON-arylesterase activity than men (*p* < 0.001), which was particularly evident in AD (*p* < 0.001) and CONTROLS (*p* < 0.001) but not in VaD (*p* = 0.202). 

In the whole cohort, there was a weak negative correlation between arylesterase activity and age (Spearman’s *r* = −0.125, *p* = 0.005), but when separated in groups there was not any significant relation. None of the other possible confounding factors (e.g., diabetes, smoking, MMSE, hypertension) had a significant relationship with arylesterase activity in the whole cohorts or in separate groups (data not shown). 

Notably, the differences observed for arylesterase activity between the groups remained significant after adjustment for age, gender, smoking, and comorbidities (ANCOVA *p* < 0.001). 

### 3.3. Serum PON-Arylesterase Activity in ADC

**Figure 2:**
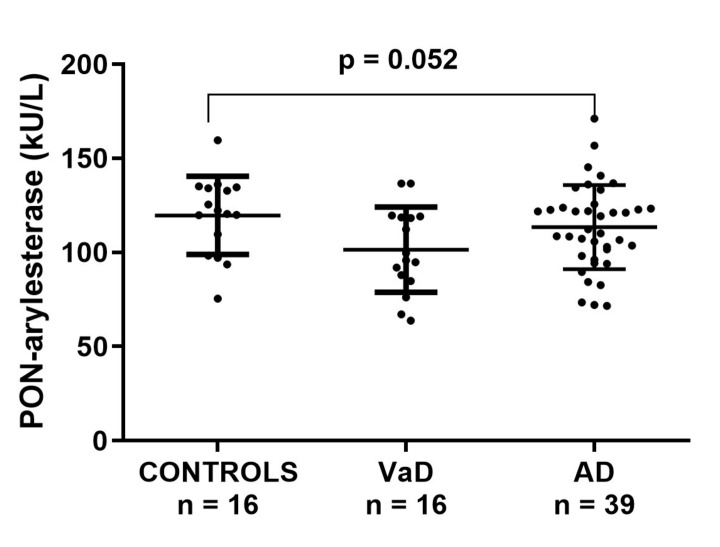
Scatter plots displaying serum PON-arylesterase activity in CONTROLS (*n* = 16), VaD (*n* = 16), and AD (*n* = 39) patients, within ADC. Statistical analysis was performed using ANOVA followed by Sidak *post-hoc* test. Abbreviations: VaD, vascular dementia; AD, Alzheimer’s disease; PON, Paraoxonase-1

Within ADC sample, serum PON-arylesterase showed a trend similar to that observed in the FC; however, only the difference between CONTROLS and VaD approached the statistical significance (post-hoc analysis, VaD vs. CONTROLS: *p* = 0.052, Figure 2). In this cohort, the PON-arylesterase mean value of the sample groups were lower compared to those of FC: CONTROLS, 104 vs. 117 kU/L; VaD, 90 vs. 101 kU/L; AD, 88 vs. 113 kU/L. Notably, only the difference between AD subsets was statistically significant (*p* < 0.001). As opposed to FC, in this cohort we did not find any significant correlation between age and PON-arylesterase activity. In addition, both sexes had comparable levels of the enzyme. The enzymatic activity was positively related to MMSE (Spearman’s *r* = 0.268, *p* < 0.05) and negatively related to hypertension (Spearman’s *r* = −0.309, *p* < 0.05). However, when separated in groups none of these correlations remained significant (data not shown). 

To explore more in depth the potential involvement of PON1 in AD and VaD, we also measured the specific activity of the enzyme (per HDL particle), by normalizing arylesterase for the levels of Apo A1 [35]. The trend for this parameter resembled that of serum PON-arylesterase, since VaD and AD displayed levels 14% and 10% lower than CONTROLS, respectively; however, no significant change was detected between the groups (Supplementary Table S2).

### 3.4. Analysis of the Potential Effect of Age on Serum PON-Arylesterase in FC and ADC

**Figure 3:**
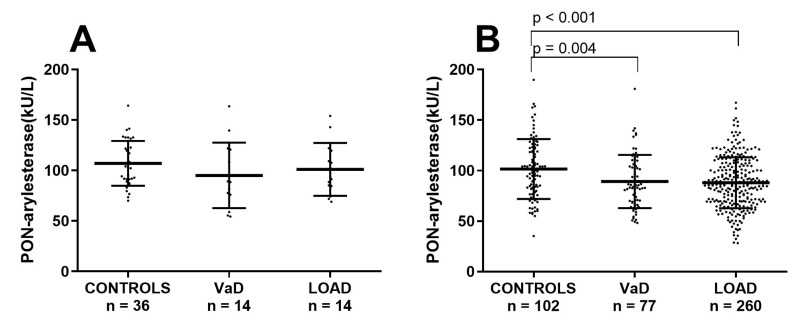
Scatter plots displaying serum PON-arylesterase activity in (**A**) CONTROLS (*n* = 36), VaD (*n* = 14), and AD (*n* = 14), within the younger subset of FC; (**B**) CONTROLS (*n* = 102), VaD (*n* = 77), and AD (*n* = 260) within the older (>71 years) subset of FC. Statistical analysis was performed using ANOVA followed by Sidak *post-hoc* test. Abbreviation: VaD, vascular dementia; LOAD, late onset Alzheimer’s disease; PON, Paraoxonase-1

One of the most evident difference in the two cohorts was the mean age of participants, with subjects within the FC being averagely 10 years older than the Amsterdam cohort. In order to check whether the discordant outcomes yielded in the two cohorts were due to age, the Ferrara’s sample was stratified in two subsets of younger or older subjects by using a cut-off of 71 years. In this way we generated a subset with CONTROLS (*n* = 36; age: 64 ± 1 years), VaD (*n* = 14, age: 67 ± 1 years) and AD (*n* = 14, age: 67 ± 1), with a mean age comparable to that of the ADC (Supplementary Table S3). As for the Amsterdam cohort, in this subsample PON-arylesterase did not significantly vary across the groups (Figure 3A).

In contrast, in the remaining older subjects from FC, PON-arylesterase exhibited a significant between groups difference, with VaD and LOAD presenting lower levels of enzymatic activity compared to CONTROLS (Figure 3B, *p* = 0.004 and *p* < 0.001, respectively, and Supplementary Table S4).

### 3.5. CSF PON-Arylesterase Activity in ADC

PON-arylesterase, Apo A1, and PON-arylesterase/ApoA1 ratio were assessed in the CSF of ADC. As summarized in Supplementary Table S5, these parameters were only slightly decreased in patients with AD or VaD compared to CONTROLS. 

Then, we evaluated the correlation between the activities measured in serum and CSF, in order to identify possible clues on the peripheral or CNS provenience of PON1 found in the CSF. As displayed in the Supplementary Figure S1A, the correlation between the two measurements was moderate (*r* = 0.390, *p* < 0.0001). Conversely, no association was detected between CSF and serum ApoA1 (Supplementary Figure S1B).

Considering that PON1 and ApoA1 are strictly related in the HDL particle, with a mutual influence on each other activity [36], we additionally evaluated the relationship between PON-arylesterase and ApoA1 in both CSF and serum. As expected, the two measurements were significantly correlated in both biological fluids, but the association was much stronger in CSF compared with serum (*r* = 0.685, *p* < 0.001 and *r* = 0.219, *p* = 0.019, respectively, data not shown).

### 3.6. Correlation of CSF Serum PON-Arylesterase, ApoA1, and PON-Arylesterase/ApoA1 Ratio with CSF t-Tau, p-tau, and Aβ1-42

**Figure 4:**
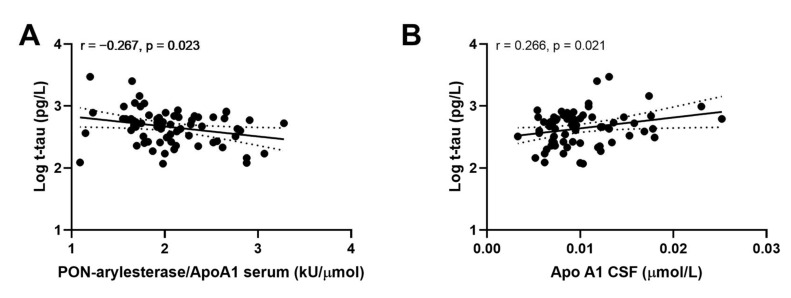
Correlation between CSF APO A1 and CSF log t-tau (**A**); serum PON-arylesterase/Apo A1 ratio and CSF log t-tau (**B**). Pearson’s correlation test was used to test the association between the variables of interest among subjects of ADC (*n* = 71). Abbreviations: Apo A1, apolipoprotein A1, CSF, cerebrovascular fluid; VaD, vascular dementia; AD, Alzheimer’s disease; PON, Paraoxonase-1

Examining the whole Amsterdam cohort, we did not find any significant correlation between CSF or serum PON-arylesterase and CSF biomarkers for AD included in this study. However, serum PON-arylesterase/ApoA1 ratio exhibited a weak inverse association with t-tau (Figure 4A, *r*: −0.267, *p*: 0.023, bootstrapped 95% CI for *r* (lower, upper): 0.085, 0.417). Of note, this correlation remained significant after adjustment for age and sex (data not shown). Moreover, significant association was also found between t-tau and CSF Apo1 (Figure 4B; *r* = 0.266; *p* = 0.021, bootstrapped 95% CI for *r* (lower, upper): 0.085, 0.417).

**Figure 5:**
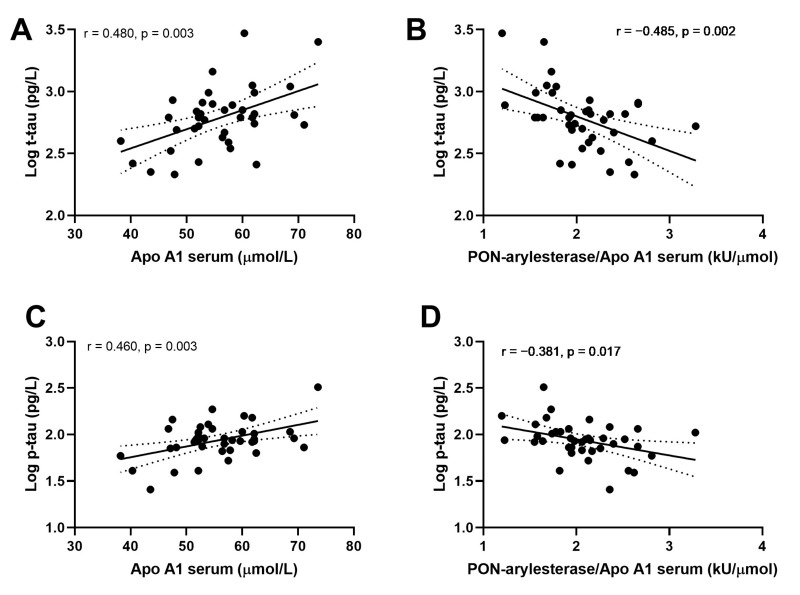
Correlation between serum APO A1 and CSF log t-tau (**A**); serum PON-arylesterase/Apo A1 ratio and CSF log t-tau (**B**); serum APO A1 and CSF log p-tau (**C**); serum PON-arylesterase/Apo A1 ratio and CSF log p-tau (**D**). Pearson’s correlation test was used to test the association between the variables of interest among AD patients of ADC (*n* = 39). Abbreviations: ApoA1, apolipoprotein A1; VaD, vascular dementia; AD, Alzheimer’s disease; PON, Paraoxonase-1

We then evaluated the associations within CONTROLS, AD and VaD groups separately. Strong correlations were detected between ApoA1 or PON-arylesterase/ApoA1 ratio, and t-tau and p-tau only in AD patients (Figure 5). In particular, serum ApoA1 was positively correlated with CSF t-tau (Figure 5A, *r* = 0.480, *p* = 0.003, bootstrapped 95% CI for *r* (lower, upper): 0.209, 0.715) and CSF p-tau (Figure 5C, *r* = 0.461, *p* = 0.003, bootstrapped 95% CI for *r* (lower, upper): 0.087, 0.699). On the contrary, the correlations with serum PON-arylesterase were both negative (vs. t-tau: Figure 5B, *r* = −0.485, *p* = 0.002, bootstrapped 95% CI for *r* (lower, upper):−0.689, −0.254; vs. p-tau: Figure 5D, *r* = −0.381, *p* = 0.017, bootstrapped 95% CI for *r* (lower, upper): −0.608, −0.126). Notably, despite serum arylesterase was not correlated with either t-tau (*r* = −0.189, *p* = 0.250) and p-tau (*r* = −0.083, *p* = 0.602), its correlation with p-tau became significant after adjustment for serum ApoA1 levels (multiple regression coefficient, beta = −0.351, *p* = 0.012).

## 4. Discussion

In the present study we demonstrated that serum PON-arylesterase activity is significantly decreased in AD and VaD patients compared to CONTROLS. When we sought to validate these findings obtained in large sample (FC) in an independent cohort (ADC), we found a similar trend, but no significant differences across the groups. Of note, this trend was also reflected in the CSF of these subjects.

The apparent discrepancy between FC and ADC data can be explained with the difference in age between the subjects of the two cohorts. Indeed, our data suggest that PON1 is altered in AD and VAD only in the elderly, but it is irrelevant in younger patients, or individuals supposedly affected by the early onset form (half of AD patients in the ADC). This notion is supported by the disappearance of association between enzyme activity and disease also in FC cohort when younger patients (71 years cut-off) were selected.

The association between serum PON1 and the presence of AD or VaD has been previously reported [9,10,11]. However, to the best of our knowledge, this is the first time that PON1 activity is assessed in the patients’ CSF, and that the association between this activity and CSF biomarkers of disease such as t-tau, p-tau, and Aβ1-42 is explored. CSF PON-arylesterase failed to show a significant association with the aforementioned biomarkers. On the other hand, serum PON-arylesterase/Apo A1 was weakly correlated with t-tau in the whole sample. A much stronger correlation was found when considering the diagnostic groups separately; from this analysis emerged that serum PON-arylesterase/Apo A1 and ApoA1 were associated with t-tau and p-tau only within the AD group.

Correlation findings give some clues of the possible mechanism underlying the impact of PON1 in the AD and VaD. The association between CSF and serum PON-arylesterase activity suggests a peripheral origin of this protein; the facts that PON1 is bound to ApoA1 in circulating HDLs and that ApoA1 in CSF originates from the periphery [37,38,39] supports our hypothesis. Besides, the moderate correlation between PON-arylesterase and ApoA1 in CSF suggests that these two proteins closely interact also in this fluid. Owing to these data, it is tempting to speculate that the decreased levels of PON-arylesterase activity observed in the large FC may reflect in CSF. This could negatively affect the still not well-defined functions of ApoA1 in CSF, which likely resemble those exerted in circulation (i.e., cholesterol transport and metabolism, vascular protection, antioxidant activity, etc.) [39]. Cumulating experimental evidence points to an additional brain-specific role of this protein: ApoA1 has been shown to bind APP and inhibit Aβ aggregation which would normally form neuritic plaques [40,41]. However, the main effects of ApoA1 could be mainly reflected on cerebrovascular Aβ accumulation [42], known as cerebral amyloid angiopathy (CAA), which is an early phenomenon in AD pathophysiology [17,43]. In line with this reasoning, lower plasma ApoA1 levels in non-demented individuals have been associated with increased risk of developing AD [44,45].

To our knowledge, the relationship between peripheral/CSF ApoA1 and biomarkers of neurodegeneration or NFT pathology has been reported only in one longitudinal study on 429 individuals with subjective cognitive decline or MCI [45]. Consistent with our results, this study showed a modest positive association between peripheral ApoA1 and CSF t-tau and p-tau only in MCI patients with high baseline levels of these two biomarkers (CSF t-tau = 506 ± 224 ng/L). On the other hand, our findings of an inverse association between CSF t-tau and p-tau and PON-arylesterase/APOA1 ratio are unprecedented, even if corroborate with the results from a recent animal study [46]. Indeed, experiments on double-knockout for ApoE and PON1 suggest that the enzyme might contribute in protecting brain from the formation of AD-related neuropathological traits, including NFT [46]. PON-arylesterase/APOA1, first introduced by Bergmeier et al. [35] reflects the activity of the enzyme per ApoA1 (surrogate marker for HDL particle) and represents a reliable measure of the biological activity of each circulating HDL [36]. High PON-arylesterase/ApoA1 ratio means, among others, more effective antioxidative protection towards ApoA1 and the other constituents of HDL, which becomes dysfunctional once oxidized [47].

The scarce evidence in this specific area, and the poor knowledge of brain tau metabolism and clearance, makes it difficult to speculate about the nature of this relationship. To advance a hypothesis in this regard, it results easier to start the reasoning from the “clinical” significance of CSF t-tau and p-tau. These two peptides are well-documented accurate prognostic biomarkers in AD. Indeed, in particular p-tau reflects the intensity of the disease, with high levels being associated with a more rapid disease progress [48,49]. Thus, for transitive propriety, PON-arylesterase/ApoA1 ratio levels could be inversely related with AD clinical progression and may possess a possible prognostic usefulness in patients with overt disease. In our opinion, this association is plausible both biologically and pathophysiologically. It is well-documented that dysfunctional HDL is implicated in the development of CVD [50,51]. In turn, peripheral vascular dysregulation leading to cerebrovascular disease has been widely proposed as the strongest pathologic factor associated with development, but also clinical progression of AD (obviously beside VaD) [52,53]. This is a piece of an emerging picture that represents AD multifactorial disease that affects both the CNS and periphery, and where abnormal systemic changes might not only develop secondary to brain dysfunction but might also affect AD progression [54,55,56].

## 5. Conclusions

Our findings suggest that AD and VaD are characterized by lower serum levels of PON-arylesterase activity compared to CONTROLS. This trend mirrors, although to a minor extent, in CSF. Intriguingly, the activity of PON1 per APOA1, that likely reflects the antioxidant protection of this apolipoprotein, emerged as a predictor of t-tau in AD patients, suggesting a potential prognostic usefulness of this parameter in affected individuals.

## Figures and Tables

**Table 1:** Principal characteristics of the Ferrara cohort (FC) and Amsterdam Dementia cohort (ADC).

Characteristics	Ferrara Cohort (FC)			Amsterdam Dementia Cohort (ADC)		
	CONTROLS (*n*: 138)	VaD (*n*: 91)	AD (*n*: 274)	CONTROLS (*n*: 16)	VaD (*n*: 16)	AD (*n*: 39)
Age (years)	75 ± 7	78 ± 7 ^a^	79 ± 5 ^a^	64 ± 6 ^c^	68 ± 6 ^c^	66 ± 6 ^c^
Female gender, No. (%)	84 (61)	54 (59)	192 (70)	8 (50)	5 (31) ^a^	16 (42) ^c^
MMSE score (/30)	27 (25–29)	21 (18–23) ^a^	20 (18–23) ^a^	29 (27–29) ^c^	24 (19–26) ^a,c^	22 (16–24) ^a^
**Medical History, No. (%)**						
Current smokers	7 (5)	8 (9)	14 (5)	2 (12)	1 (6)	5 (21)
Hypertension	85 (62)	64 (70)	178 (65)	1 (7) ^c^	13 (80) ^a^	8 (19) ^c^
Diabetes	19 (14)	25 (28) ^a^	38 (14) ^b^	2 (13)	4 (21)	4 (11)

Continuous variables are expressed as mean ± SEM or median (interquartile range); ^a^ *p* < 0.05 vs. CONTROLS; ^b^ *p* < 0.05 vs. VaD; ^c^ *p* < 0.05 vs. the same group in FC. Abbreviations: CVD, cardiovascular diseases; MMSE, mini mental state examination; VaD, vascular dementia; AD, Alzheimer’s disease. Statistical analysis was performed using ANOVA followed by Sidak post-hoc test.

## Supplementary Materials

The following are available online at https://www.mdpi.com/2076-3921/9/5/456/s1, Figure S1: Correlation between serum and CSF PON-arylesterase and between serum and CSF APO A1; Table S1: CSF biomarker values and MRI findings in Amsterdam Dementia cohort; Table S2: Serum Apo A1 and PON-arylesterase/Apo A1 ratio in ADC; Table S3: Age and serum PON-arylesterase activity in younger (<71 years) subjects of FC; Table S4: Age and serum PON-arylesterase in older subjects (>71 years) of FC; Table S5: PON-arylesterase, APO A1 and PON-arylesterase/Apo A1 ratio in CSF of ADC.
