# Supplementary material for: Arylesterase Activity of Paraoxonase-1 in Serum and Cerebrospinal Fluid of Patients with Alzheimer’s Disease and Vascular Dementia
Source: Antioxidants (Basel). 2020 May 25;9(5):456. doi: 10.3390/antiox9050456 (PMC7278748; doi:10.3390/antiox9050456)
Supplement: Supplementary file 1 [file antioxidants-09-00456-s001.pdf]

Supplementary Table S1. CSF biomarker values and MRI findings in Amsterdam dementia cohort.

Characteristic	Controls (*n*: 16)	VaD (*n*: 16)	AD (*n*: 39)
**CSF biomarkers, pg/mL**			
**CSF Aβ1-42**	972 ± 249	815 ± 251	637 ± 146 [a,b]
**CSF t-tau**	316 ± 160	396 ± 192	725 ± 540 [a,b]
**CSF p-tau**	55 ± 18	53 ± 22	97 ± 49 [a,b]
**MRI characteristics, No (%)**			
**WML> 1**	10 (60)	16 (100) [a]	30 (7395) [a,b]
**Lacunes present**	0 (0)	13 (81) [a]	2 (5)
**MTA> 1**	1 (3)	14 (87) [a]	18 (45) [a,b]

Continuous variables are expressed as mean ± SEM or median (interquartile range); categorical variable are expressed as percentage within group; [a] $p < 0.05$ vs CONTROLS; [b] $p < 0.05$ vs VaD. Abbreviations: CVD, cardiovascular diseases; MMSE, Mini Mental State Examination; WMLs white mass lesions; MTA, medial temporal lobe atrophy (MTA). Statistical analysis was performed using ANOVA followed by Sidak post hoc test

Supplementary Table S2. Serum Apo A1 and PON-arylesterase/Apo A1 ratio in ADC

Characteristic	CONTROLS (*n*: 16)	VAD (*n*: 16)	AD (*n*: 39)
**Apo A1 (μmol/L)**	55 ± 9	53 ± 10	55 ± 8
**PON-Arylesterase/Apo A1 (kU/μmol)**	2.2 ± 0.4	1.9 ± 0.5	2.0 ± 0.4

Continuous variables are expressed as mean ± SEM. Statistical analysis was performed using ANOVA followed by Sidak post hoc test

Supplementary Table S3. Age and serum PON-arylesterase activity in younger (< 71 years) subjects of FC

Characteristic	CONTROLS (*n*: 36)	VaD (*n*: 14)	AD (*n*: 14)
**Age (years)**	64 ± 5	67 ± 4	67± 3
**PON-Arylesterase (kU/L)**	106 ± 23	95 ± 32	101 ± 26

Continuous variables are expressed as mean ± SEM. Statistical analysis was performed using ANOVA followed by Sidak post hoc test

Supplementary Table S4. Age and serum PON-arylesterase in older subjects (> 71 years) of FC

Characteristic	CONTROLS (*n*: 102)	VaD (*n*: 77)	LOAD (*n*: 260)
**Age (years)**	79 ± 4	80 ± 5	80 ± 5
**PON-Arylesterase (kU/L)**	101 ± 28	88 ± 29[a]	87 ± 16[a]

Continuous variables are expressed as mean ± SEM. Statistical analysis was performed using ANOVA followed by Sidak post hoc test. [a] $p < 0.05$ vs CONTROLS.

Supplementary Table S5. PON-arylesterase, APO A1 and PON-arylesterase/Apo A1 ratio in CSF of ADC

Characteristic	CONTROLS (*n*: 16)	VaD (*n*: 16)	LOAD (*n*: 39)
**PON-Arylesterase (kU/L)**	0.09 ± 0.02	0.07 ± 0.03	0.07 ± 0.04
**Apo A1 (μmol/L)**	0.03 ± 0.01	0.02 ± 0.00	0.03 ± 0.01
**PON-Arylesterase/Apo A1 (U/μmol)**	2.9 ± 1.0	2.7 ± 1.0	2.6 ± 0.8

Continuous variables are expressed as mean ± SEM. Statistical analysis was performed using ANOVA followed by Sidak post hoc test

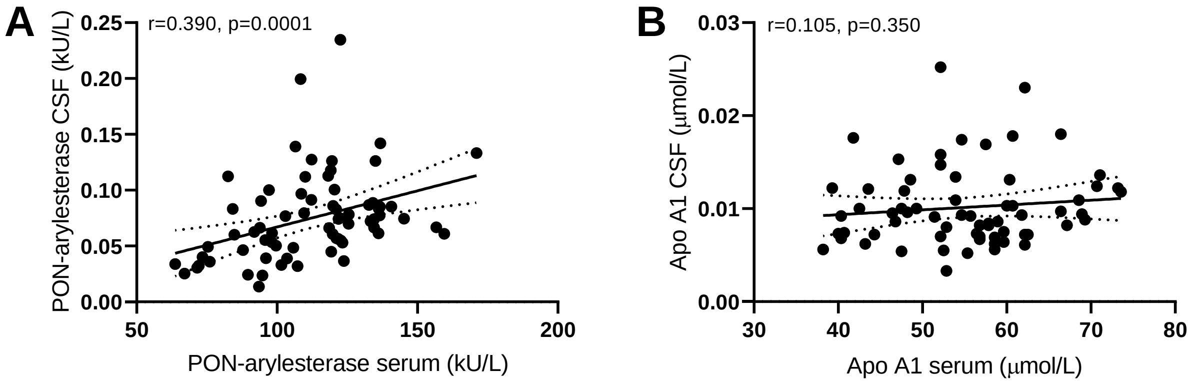

Supplemental Figure S1 Correlation between serum and CSF PON-arylesterase (**A**), and between serum and CSF APO A1 (**B**). Pearson's correlation test was used to test the association between the variables of interest among subjects of ADC (n = 71).
